# The lymphatic system and COVID-19 vaccines

**DOI:** 10.3389/fimmu.2022.1041025

**Published:** 2022-10-20

**Authors:** Masayuki Miyasaka

**Affiliations:** Immunology Frontier Research Center, Osaka University, Suita, Japan

**Keywords:** COVID-19, lymphatics, lymph node, mRNA vaccines, SARS-CoV-2

## Abstract

Understanding the precise mechanism of vaccine-induced protection and the immune correlates of protection against coronavirus disease 2019 (COVID-19) is crucially important for developing next-generation vaccines that confer durable and protective immunity against COVID-19. Similar factors are also important for other infectious diseases. Here, I briefly summarize the mechanism of action of the currently used COVID-19 mRNA vaccines from the viewpoint of the function of the lymphatic system.

## COVID-19 and its vaccines

Coronavirus disease 2019 (COVID-19) emerged suddenly in December 2019 in Wuhan, China. It is caused by a novel single-stranded RNA virus, severe acute respiratory syndrome coronavirus 2 (SARS-CoV-2) (1, 2). To date, this virus has infected almost 600 million people, which has resulted in more than 6 million deaths globally, as of the end of August 2022 (3).

COVID-19 spreads from person to person mainly *via* the respiratory route, specifically through airborne macro- and micro-droplets (or aerosols) emitted by virus carriers. Two types of cell surface proteins are involved in the SARS-CoV-2 infection of human cells. One is angiotensin-converting enzyme (ACE2), to which the viral spike (S) protein of SARS-CoV-2 binds. It is expressed in varying degrees by a wide range of tissue types (4). The other cell surface protein is transmembrane serine protease 2 (TMPRSS2), which proteolytically cleaves the viral S protein, allowing the virus to enter and infect human cells (5). TMPRSS2 is also expressed by a number of cell types. Owing to the wide expression of both ACE2 and TMPRSS2, SARS-CoV-2 targets not only respiratory epithelial cells but also a variety of other cell types, including intestinal epithelial cells, blood vascular endothelial cells, adipocytes, cardiomyocytes, and neuronal cells, which enables this virus to enter and damage numerous tissues in the body. Such behavior is in marked contrast with that of other respiratory viruses, such as influenza virus, which target mainly the cells of the respiratory tract.

At present, the most effective prevention measure against COVID-19 is vaccination (6). Two different mRNA vaccines, namely the Pfizer (BNT162b2) vaccine and the Moderna (mRNA-1273) vaccine, are being widely used. A two-dose series of either of these mRNA vaccines has been shown to markedly reduce the risk of not only symptomatic infection but also hospitalization and mortality from COVID-19 (7, 8). Booster immunizations can further enhance the protection from the primary series vaccination that otherwise wanes over time (9–11).

These mRNA vaccines aim to induce immunity against the S protein of SARS-CoV-2. This protein consists of an apical S1 subunit, which contains an N-terminal domain (NTD) and a receptor-binding domain (RBD), and a membrane-proximal S2 subunit; the S1 subunit binds to the ACE2 on human cells, whereas the S2 subunit is responsible for the fusion of viral and cellular membranes (12). These subunits are functionally indispensable for viral binding to and entry into human cells, and correspondingly, antibodies and T cells generated against this protein have been shown to have the capacity to inhibit SARS-CoV-2 infection of human cells (13).

Several strategies have been adopted to increase the immunogenicity of the COVID-19 mRNA vaccines (14–16). First, all uridine residues in the S protein mRNA have been replaced with N1-methyl pseudouridine residues; this strategy helps the exogenous RNA to evade recognition by innate immunity receptors such as Toll-like receptors (TLRs) (17). Second, a 5′-cap structure, a polyadenosine (poly-A) tail, and untranslated regions (UTRs) have all been included in the vaccine mRNA because these components facilitate the translation of viral proteins and provide protection from the ubiquitous exonucleases in the body. Third, to reduce mRNA degradation after vaccine administration and to promote mRNA delivery into cells, the mRNA has been encapsulated in biodegradable lipid nanoparticles consisting of cationic lipids, phospholipids, cholesterol, and polyethylene glycol-containing lipids. These lipid nanoparticles also act as an adjuvant component of the mRNA vaccines, inducing interleukin (IL)-6 cytokine production, which leads to the strong induction of T follicular helper T cells, germinal center B cells, and memory B-cell responses in the draining lymph nodes (LNs) of vaccine recipients (18). Fourth, to make the S protein more immunogenic, the furin cleavage site between S1 and S2 has been removed, and two amino acid residues in S2 have been substituted with proline (K986P/V987P), which together allow the formation of a relatively stable S protein trimer when the mRNA is incorporated and translated into proteins in target cells (16).

Because of these modifications, the COVID-19 mRNA vaccines are highly immunogenic, inducing strong serum neutralizing antibody responses in most individuals, whereas infection with SARS-CoV-2 produces more variable neutralizing antibody responses (19, 20); in both cases, epitopes that overlap the ACE2-binding site in the RBD are targeted strongly by the generated antibodies. While neutralizing antibodies are a principal host defense mechanism against viral invasion, the mRNA COVID-19 vaccines also activate T cells, which play an important role in limiting disease severity and duration, as described below.

## COVID-19 mRNA vaccines are delivered to the draining LNs *via* the lymphatics to induce immune priming

Another remarkable feature of the COVID-19 mRNA vaccines is that they can be readily incorporated into the lymphatic system. This is mainly because these vaccines are encapsulated in lipid nanoparticles approximately 100 nm in diameter. Lipids are known to be preferentially transported to the lymphatic system (21), and particles with a size range of 10 to 100 nm readily enter into the lymphatics (22).

The COVID-19 mRNA vaccines are administered intramuscularly (into the deltoid muscle in most cases), and muscles are rich in both blood vessels and lymphatics. Because these vaccines are preferentially transported into the lymphatics as described above, following their intramuscular injection, they are swiftly delivered to the lymph nodes that drain the muscle. Studies with experimental animals revealed that vaccine transport after intramuscular injection is restricted mainly to the local LNs that drain the injection site and that the vaccine is not readily disseminated systemically (23–25), unless non-physiologically large doses are injected. Indeed, a PET CT (positron emission tomography and computed tomography) study using [^18^F]FDG in human volunteers confirmed that prominent vaccine-associated LN swelling occurs selectively in the axillary and supraclavicular LNs of the ipsilateral side in relation to the vaccine injection site (26). Another study found that the LN swelling often persisted for more than 12 weeks (27). These LNs are the sites where the cells of the innate and adaptive immune systems interact.

In the draining LNs, although most phagocytic cells can internalize the mRNA vaccine, macrophages within the subcapsular sinus and dendritic cells (DCs) within the interfollicular area are the main groups that abundantly take up specific antigens. However, it is mainly DCs that exhibit high levels of translation of the vaccine mRNA and the upregulation of key co-stimulatory molecules (CD80 and CD86) essential for efficient antigen presentation to naïve T cells (24). These DCs subsequently display an upregulation of type I interferon (IFN)-inducible genes, leading to the production of a number of cytokines/chemokines required for antigen-specific stimulation of naïve T and B lymphocytes.

Within DCs, the majority of the S protein *in situ* generated from the vaccine mRNA is degraded in endosome-derived proteasomes, and some of the generated peptides are transported to the endoplasmic reticulum, where they are loaded onto class I major histocompatibility complexes (MHCs). Additionally, part of the S protein is produced and secreted as an exogenous protein by the cells that took up the mRNA. It is then taken up by professional antigen-presenting cells, such as DCs, in which the protein is processed and presented in class II MHCs. Peptide presentation by class II MHCs also occurs on the surface of DCs after their uptake of cell debris containing the vaccine-encoded protein. These peptide-loaded MHC class I and class II molecules on the surface of DCs subsequently present their antigens to antigen-specific CD4^+^ and CD8^+^ T cells, respectively (28, 29).

Detectable protein production can be found for up to ten days at the site of injection after the intramuscular injection of a COVID-19 mRNA vaccine (30). While some muscle cells endocytose the vaccine components, they are unable to effectively present the encoded antigen to the lymphoid system because they lack expression of the key co-stimulatory molecules (CD80 and CD86) essential for efficient antigen presentation to antigen-inexperienced naïve lymphocytes (31). Therefore, mRNA translation in muscle cells does not seem to play a major role in the induction of protective immune responses against SARS-CoV-2.

The innate immune activation by mRNA vaccines sometimes occurs in excess, resulting in adverse reactions, which can include pain, swelling, and redness at the local injection site (32, 33). Systemic side effects, such as allergy, chills, fever, and headache, are also often observed in recipients of COVID-19 mRNA vaccines. The occurrence of side effects from vaccination with a COVID-19 mRNA vaccine is likely a consequence of the intrinsic adjuvant activity of the current lipid nanoparticle formulation, which induces the robust production of inflammatory cytokines, such as IL-6, by the innate immune system (18); such a response tends to be more prominent in young people. Nevertheless, it should be noted that lipid nanoparticle-mediated IL-6 production is critical for the induction of follicular helper T cells in response to mRNA vaccination and that the protective vaccine effect is low in the absence of follicular helper T-cell proliferation (18).

## COVID-19 mRNA vaccines activate adaptive immunity in the draining LNs

The transport of vaccine antigen to the draining LNs is crucial for the initiation of systemic immune responses against the relevant pathogen by T and B cells (23). Previous studies have indicated that protective immunity against COVID-19 is mediated by both key arms of the adaptive immune system, i.e., humoral immunity and cellular immunity (34–37), and that antibody and T-cell responses tend to work in a compensatory manner to provide protection (38). Humoral immunity generates antibodies and memory B cells, while cellular immunity leads to the activation of antigen-specific helper CD4^+^ T cells and cytotoxic CD8^+^ T cells (13), which both occur in the draining LNs (39, 40).

Antibodies are particularly important in early protection against viral infection because they block infection by binding the virus and preventing viral entry into host cells. Additionally, memory B cells can produce high-affinity neutralizing antibodies upon re-encounter with the same virus. T cells, in contrast, are unable to block the initial infection process. Instead, they become activated upon antigen presentation by DCs, after which they proliferate to limit viral replication and spread. The CD4^+^-dependent cytotoxic CD8^+^ T-cell response is particularly important, and when the population of these cells expands within 7 days of symptoms and peaks at 14 days, viruses are cleared effectively (41), leading to mild rather than severe disease (42). Consistently, most individuals with resolved infections show robust and broad enhanced T-cell responses against multiple regions of SARS-CoV-2 (43, 44). Vaccine-induced T-cell responses have good cross-reactivity to the many variants of SARS-CoV-2 that have emerged recently (45, 46).

Turner et al. (40) examined antigen-specific B-cell responses in the peripheral blood and draining LNs of Pfizer mRNA vaccine recipients. They found that the populations of circulating immunoglobulin (Ig)G- and IgA-secreting plasmablasts that target the SARS-CoV-2 S protein peaked 1 week after the second immunization, declining thereafter, and that plasma neutralizing IgG antibody titers also showed similar kinetics. These researchers also conducted a needle biopsy of the draining LNs and examined cellular responses *in situ*. They found that S-binding germinal centers increased in frequency after the second immunization and that these germinal centers persisted at high frequency for 15 weeks. S-binding plasmablasts were also abundant in the draining LNs and persisted there even after they became undetectable in the peripheral blood. The majority of monoclonal antibodies generated from the germinal center-derived B cells strongly reacted with the S protein RBD as expected, while some reacted with the S proteins of the human betacoronaviruses OC43 and HKU1. These results indicate that the S-binding germinal centers recruited not only naïve B cells targeting unique epitopes within the SARS-CoV-2 S protein but also pre-existing memory B cells directed against epitopes conserved among human betacoronaviruses.

Nevertheless, patients with X-linked agammaglobulinemia have been reported to recover from COVID-19 (47), indicating that antibody-independent mechanism(s) also plays an important role in virus clearance.

## COVID-19 mRNA vaccines induce memory responses

Upon immunization with multiple doses of COVID-19 mRNA vaccine, SARS-CoV-2-reactive memory B cells appear systemically and persist for more than 6 months (48), subsequent to the appearance of the S protein-binding germinal centers in the draining LNs (40). Booster vaccinations increase the frequency of these memory B cells, inducing them to produce antibodies with significantly higher potency and breadth compared with the antibodies obtained after the second COVID-19 vaccine dose (49). Additionally, these antibodies are capable of neutralizing SARS-CoV-2 variants of multiple lineages (50), reducing the escape by mutant virus strains relative to ancestral virus (50, 51).

## What should be improved with mRNA vaccines?

For non-COVID-19 respiratory virus infections, pre-existing immunity induced by natural infection or vaccination has been reported to induce antibody-dependent enhancement (ADE) of disease in some instances (52). Indeed, infectivity-enhancing antibodies have been found in a proportion of COVID-19 patients, and these antibodies tend to bind to a specific site of the SARS-CoV-2 S protein (53, 54). Nevertheless, the production of such antibodies was associated with the production of neutralizing antibodies, and enhancing antibodies did not induce ADE when neutralizing antibodies were present at high levels *in vitro* (53). Because of these observations, ADE continues to be a substantial concern in the development of COVID-19 vaccines. To the best of my knowledge, however, there is currently no evidence of vaccine-associated enhanced respiratory disease occurring following immunization with the COVID-19 mRNA vaccines currently in use, although this issue warrants further investigation because a new generation of mRNA vaccines is now becoming available.

In addition to the potential ADE issue, one major problem with the current COVID-19 mRNA vaccines, although they are highly effective at reducing the incidence and severity of SARS-CoV-2 infection, is the waning of their efficacy with increasing time since the second dose (11, 55). However, this issue seems to be more related to the pathogen than it is to the specific vaccines. A longitudinal study on SARS-CoV-2 reinfection indicates that, with natural infection, the mean time by which there is a 5% cumulative risk of reinfection was only approximately 140 days post-symptom onset, which is less than half of the mean time to a cumulative 5% risk of breakthrough infection following vaccination with Pfizer or Moderna mRNA vaccine (both required approximately 350 days post-vaccination to reach this point) (20). These results indicate that the immunity induced by SARS-CoV-2 natural infection begins to wane after several months post-infection and that the immunity from natural infection lasts for a much shorter length of time compared with the duration of vaccine-mediated immunity.

While inducing durable immunity against SARS-CoV-2 does not appear to be an easy task, there may be at least a couple of ways of making the COVID-19 vaccine response more durable. One is to enhance the innate immune mechanism through which mRNA vaccines stimulate protective antibody responses. The other is to enhance the adaptive immune mechanism, particularly to enhance T-cell memory cell responses. Regarding the former, using other types of lipid particles or adding an appropriate adjuvant to the mRNA vaccines is a possibility, but this approach carries the possibility of increasing the incidence of adverse reactions as well. Regarding the latter, attempting to stimulate T cells with not only the S protein but also the matrix (M) protein and nucleocapsid (N) protein is also a possibility (56). The inclusion of other viral structural protein epitopes may strengthen T-cell responses, thereby amplifying and diversifying the induced antibody response against SARS-CoV-2.

While the current COVID-19 mRNA vaccines are highly protective against the development of severe disease, they elicit limited immune responses in the respiratory tract (57). Vaccines targeting the respiratory mucosa are now under development with the hope of inducing robust and durable immunity specifically in the respiratory tract. In particular, a mucosal booster vaccine may be an effective strategy to achieve more robust and long-lasting immunity against SARS-CoV-2.

## Conclusion

COVID-19 vaccine development is moving forward at unprecedented speed, but several challenges remain. For example, the current COVID-19 mRNA vaccines are less effective at blocking infection by the newly arising SARS-CoV-2 variants, although protection against severe disease remains well preserved. Additionally, only relatively weak immune responses are induced in the mucosa of many vaccine recipients, and even when such responses are successfully induced, they tend to wane rapidly. Furthermore, the virus continues evolving ways to evade our immune response. Thus, there is an urgent need to develop pan-coronavirus vaccines that can target not only the current SARS-CoV-2 variants but also future variants. The development of mucosal vaccines that are delivered across mucosal barriers may also present a promising strategy to promote durable protection against SARS-CoV-2 at the mucosal level.

## Author contributions

The author confirms being the sole contributor of this work and has approved it for publication.

## Acknowledgments

I thank Katie Oakley, PhD, from Edanz (https://jp.edanz.com/ac) for editing a draft of this manuscript.

## Conflict of interest

The author declares that the research was conducted in the absence of any commercial or financial relationships that could be construed as a potential conflict of interest.

## Publisher’s note

All claims expressed in this article are solely those of the authors and do not necessarily represent those of their affiliated organizations, or those of the publisher, the editors and the reviewers. Any product that may be evaluated in this article, or claim that may be made by its manufacturer, is not guaranteed or endorsed by the publisher.

## References

[1] Wiersinga WJ Rhodes A Cheng AC Peacock SJ Prescott HC. Pathophysiology, transmission, diagnosis, and treatment of coronavirus disease 2019 (COVID-19): A review. JAMA (2020) 344:782–93. doi: 10.1001/jama.2020.12839 32648899

[2] Hu B Guo H Zhou P Shi Z-L. Characteristics of SARS-CoV-2 and COVID-19. Nat Rev Microbiol (2021) 19:141. doi: 10.1038/s41579-020-00459-7 33024307 PMC7537588

[3] Available at: https://www.worldometers.info/coronavirus/.

[4] Kuba K Yamaguchi T Penninger JM. Angiotensin-converting enzyme 2 (ACE2) in the pathogenesis of ARDS in COVID-19. Front Immunol (2021) 12:732690. doi: 10.3389/fimmu.2021.732690 35003058 PMC8727358

[5] Hoffmann M Kleine-Weber H Schroeder S Krüger N Herrler T Erichsen S. SARS-CoV-2 cell entry depends on ACE2 and TMPRSS2 and is blocked by a clinically proven protease inhibitor. Cell (2020) 181:271–280.e8. doi: 10.1016/j.cell.2020.02.052 32142651 PMC7102627

[6] Barouch DH. Covid-19 vaccines — immunity, variants, boosters. New Engl J Med (2022) 387:1–3. doi: 10.1056/NEJMra2206573 36044620 PMC9454645

[7] Polack FP Thomas SJ Kitchin N Absalon J Gurtman A Lockhart S. Safety and efficacy of the BNT162b2 mRNA covid-19 vaccine. New Engl J Med (2020) 387:2603–15. doi: 10.1056/NEJMoa2034577 PMC7745181 33301246

[8] Baden LR El Sahly HM Essink B Kotloff K Frey S Novak R. Efficacy and safety of the mRNA-1273 SARS-CoV-2 vaccine. New Engl J Med (2021) 384:403–16. doi: 10.1056/NEJMoa2035389 PMC7787219 33378609

[9] Tregoning JS Flight KE Higham SL Wang Z Pierce BF. Progress of the COVID-19 vaccine effort: Viruses, vaccines and variants versus efficacy, effectiveness and escape. Nat Rev Immunol (2020) 21:626. doi: 10.1038/s41577-021-00592-1 PMC8351583 34373623

[10] Chaudhary N Weissman D Whitehead KA. mRNA vaccines for infectious diseases: Principles, delivery and clinical translation. Nat Rev Drug Discovery (2021) . 20:817–38. doi: 10.1038/s41573-021-00283-5 PMC8386155 34433919

[11] Feikin DR Higdon MM Abu-Raddad LJ Andrews N Araos R Goldberg Y. Duration of effectiveness of vaccines against SARS-CoV-2 infection and COVID-19 disease: Results of a systematic review and meta-regression. Lancet (2022) 399:924–44. doi: 10.1016/S0140-6736(22)00152-0 PMC8863502 35202601

[12] Harvey WT Carabelli AM Jackson B Gupta RK Thomson EC Harrison EM. SARS-CoV-2 variants, spike mutations and immune escape. Nat Rev Microbiol (2021) 19:409–24. doi: 10.1038/s41579-021-00573-0 PMC8167834 34075212

[13] Wherry EJ Barouch DH T. Cell iimmunity to COVID-19 vaccines. Science (2022) . 377:821–2. doi: 10.1126/science.add2897 35981045

[14] Bettini E Locci M. SARS-CoV-2 mRNA vaccines: Immunological mechanism and beyond. Vaccines (2021) 9:147–65. doi: 10.3390/vaccines9020147 PMC7918810 33673048

[15] Hogan MJ Norbert P. mRNA vaccines in the COVID-19 pandemic and beyond. Ann Rev Med (2022) . 73:17–39. doi: 10.1146/annurev-med-042420-112725 34669432

[16] Gohda E. Effectiveness of and immune responses to SARS-CoV-2 mRNA vaccines and their mechanisms. J Disast Res (2022) 17:7–20. doi: 10.20965/jdr.2022.p0007

[17] Karikó K Buckstein M Ni H Weissman D. Suppression of RNA recognition by toll-like receptors: the impact of nucleoside modification and the evolutionary origin of RNA. Immunity (2005) 23:165–75. doi: 10.1016/j.immuni.2005.06.008 16111635

[18] Alameh MG Tombácz I Bettini E Lederer K Sittplangkoon C Wilmore JR. Lipid nanoparticles enhance the efficacy of mRNA and protein subunit vaccines by inducing robust T follicular helper cell and humoral responses. Immunity (2021) . 54:2877–2892.e7. doi: 10.1016/j.immuni.2021.11.001 34852217 PMC8566475

[19] Hall V Foulkes S Insalata F Kirwan P Saei A Atti A. Protection against SARS-CoV-2 after covid-19 vaccination and previous infection. New Engl J Med (2022) 386:1207–20. doi: 10.1056/NEJMoa2118691 PMC8908850 35172051

[20] Townsend JP Hassler HB Sah P Galvani AP Dornburg A. The durability of natural infection and vaccine-induced immunity against future infection by SARS-CoV-2. Proc Natl Acad Sci USA (2022) 119:e2204336119. doi: 10.1073/pnas.2204336119 35858382 PMC9351502

[21] Dixon JB. Mechanisms of chylomicron uptake into lacteals. Ann NY Acad Sci (2010) . 1207 Suppl:E52–7. doi: 10.1111/j.1749-6632.2010.05716.x PMC3132563 20961306

[22] Lee J Kim D Byun J Wu Y Park J Oh YK. *In vivo* fate and intracellular trafficking of vaccine delivery systems. Adv Drug Delivery Res (2022) 186:114325. doi: 10.1016/j.addr.2022.114325 PMC9085465 35550392

[23] Liang F Lindgren G Sandgren KJ Thompson EA Francica JR Seubert A. Vaccine priming is restricted to draining lymph nodes and controlled by adjuvant-mediated antigen uptake. Sci Transl Med (2017) 9:eaal2094. doi: 10.1126/scitranslmed.aal2094 28592561

[24] Liang F Lindgren G Lin A Thompson EA Ols S Röhss J. Efficient targeting and activation of antigen-presenting cells *In vitro* and *In vivo* after modified mRNA vaccine administration in rhesus macaques. Mol Ther (2017) . 25:2635–47. doi: 10.1016/j.ymthe.2017.08.006 PMC5768558 28958578

[25] Lindsay KE Bhosle SM Zurla C Beyersdorf J Rogers KA Vanover D. Visualization of early events in mRNA vaccine delivery in non-human primates *via* PET–CT and near-infrared imaging. Nat BioMed Engin (2019) . 3:371–80. doi: 10.1038/s41551-019-0378-3 30936432

[26] Cohen D Krauthammer SH Wolf I Even-Sapir E. Hypermetabolic lymphadenopathy following administration of BNT162b2 mRNA covid-19 vaccine: Incidence assessed by [18F]FDG PET-CT and relevance to study interpretation. Eur J Nucl Med Mol Imaging (2021) . 48:1854–63. doi: 10.1007/s00259-021-05314-2 PMC8003894 33774684

[27] Ha SM Chu AJ Lee J Kim S-Y Lee SH Yoen H. US Evaluation of axillary lymphadenopathy following COVID-19 vaccination: A prospective longitudinal study. Radiol (2022) 305:46. doi: 10.1148/radiol.220543 PMC9096883 35471107

[28] Rijkers GT Weterings N Obregon-Henao A Lepolder M Dutt TS van Overveld FJ. Antigen presentation of mRNA-based and virus-vectored SARS-CoV-2 vaccines. Vaccines (2021) . 9:848–65. doi: 10.3390/vaccines9080848 PMC8402319 34451973

[29] Heinz FX Stiasny K. Distinguishing features of current COVID-19 caccines: Knowns and unknowns of antigen presentation and modes of action. NPJ Vaccines (2021) . 6:104. doi: 10.1038/s41541-021-00369-6 34400651 PMC8368295

[30] Cagigi A Loré K. Immune responses induced by mRNA vaccination in mice, monkeys and humans. Vaccines (2021) . 9:61–75. doi: 10.3390/vaccines9010061 33477534 PMC7831080

[31] Behrens L Kerschensteiner M Misgeld T Goebels N Wekerle H Hohlfeld R. Human muscle cells express a functional costimulatory molecule distinct from B7.1 (CD80) and B7.2 (CD86) *in vitro* and in inflammatory lesions. J Immunol (1998) 161:5943–51. 9834075

[32] Awaya T Moroi M Enomoto Y Kunimasa T Nakamura M. What should we do after the COVID-19 vaccination? vaccine-associated diseases and precautionary measures against adverse reactions. Vaccines (2022) . 10:866–79. doi: 10.3390/vaccines10060866 PMC9228524 35746474

[33] Rosa Duque JS Wang X Leung D Cheng SMS Cohen CA Mu X. Immunogenicity and reactogenicity of SARS-CoV-2 vaccines BNT162b2 and CoronaVac in healthy adolescents. Nat Comm (2022) . 13:3700. doi: 10.1038/s41467-022-31485-z PMC9240007 35764637

[34] Ni L Ye F Cheng M-L Feng Y Deng Y-Q Zhao H. Detection of SARS-CoV-2-Specific humoral and cellular immunity in COVID-19 convalescent individuals. Immunity (2020) . 52:971–7. doi: 10.1016/j.immuni.2020.04.023 PMC7196424 32413330

[35] Li K Huang B Wu M Zhong A Li L Cai Y. Dynamic changes in anti-SARS-CoV-2 antibodies during SARS-CoV-2 infection and recovery from COVID-19. Nat Comm (2020) 11:6044. doi: 10.1038/s41467-020-19943-y PMC7699636 33247152

[36] Chia WN Zhu F Ong SWX Young BE Fong S-W Le Bert N. Dynamics of SARS-CoV-2 neutralising antibody responses and duration of immunity: A longitudinal study. Lancet Microbe (2021) . 2:e240–9. doi: 10.1016/S2666-5247(21)00025-2 PMC7987301 33778792

[37] Miyasaka M. COVID-19 and immunity: Quo vadis? Int Immunol (2021) . 33:507–13. doi: 10.1093/intimm/dxab008 PMC7928925 33569606

[38] Menges D Zens KD Ballouz T Caduff N Llanas-Cornejo D Aschmann HE. Heterogenous humoral and cellular immune responses with distinct trajectories post-SARS-CoV-2 infection in a population-based cohort. Nat Comm (2022) . 13:4855. doi: 10.1038/s41467-022-32573-w PMC9386650 35982045

[39] Mudd PA Minervina AA Pogorelyy MV Turner JS Kim W Kalaidina E. SARS-CoV-2 mRNA vaccination elicits a robust and persistent T follicular helper cell response in humans. Cell (2022) . 185:603–615.e15. doi: 10.1016/j.cell.2021.12.026 35026152 PMC8695127

[40] Turner JS O’Halloran JA Kalaidina E Kim W Schmitz AJ JQ Z. SARS-CoV-2 mRNA vaccines induce persistent human germinal centre responses. Nature (2021) . 596:109–13. doi: 10.1038/s41586-021-03738-2 PMC8935394 34182569

[41] Notarbartolo S Ranzani V Bandera A Gruarin P Bevilacqua V Putignano AR. Integrated longitudinal immunophenotypic, transcriptional and repertoire analyses delineate immune responses in COVID-19 patients. Sci Immunol (2021) 6:eabg5021. doi: 10.1126/sciimmunol.abg5021 34376481

[42] Bergamaschi L Mescia F Turner L Hanson AL Kotagiri P Dunmore BJ. Longitudinal analysis reveals that delayed bystander CD8^+^ T cell activation and early immune pathology distinguish severe COVID-19 from mild disease. Immunity (2021) . 54:1257–75. doi: 10.1016/j.immuni.2021.05.010 PMC8125900 34051148

[43] Sekine T Perez-Potti A Rivera-Ballesteros O Strålin K Gorin JB Olsson A. Robust T cell immunity in convalescent individuals with asymptomatic or mild COVID-19. Cell (2020) . 183:158–168.e14. doi: 10.1016/j.cell.2020.08.017 32979941 PMC7427556

[44] Peng Y Mentzer AJ Liu G Yao X Yin Z Dong D. Broad and strong memory CD4^+^ and CD8^+^ T cells induced by SARS-CoV-2 in UK convalescent individuals following COVID-19. Nat Immunol (2020) . 21:1336–45. doi: 10.1038/s41590-020-0782-6 PMC7611020 32887977

[45] Goel RR Apostolidis SA Painter MM Mathew D Pattekar A Kuthuru O. Distinct antibody and memory b cell responses in SARS-CoV-2 naïve and recovered individuals following mRNA vaccination. Sci Immunol (2021) 6:eabi6950. doi: 10.1126/sciimmunol.abi6950 33858945 PMC8158969

[46] Dan JM Mateus J Kato Y Hastie KM Yu ED Faliti CE. Immunological memory to SARS-CoV-2 assessed for up to 8 months after infection. Science (2021) 371:eabf4063. doi: 10.1126/science.abf4063 33408181 PMC7919858

[47] Soresina A Moratto D Chiarini M Paolillo C Baresi G Focà E. Two X-linked agammaglobulinemia patients develop pneumonia as COVID-19 manifestation but recover. Pediatr Allergy Immunol (2020) 31:565–9. doi: 10.1111/pai.13263 PMC7264678 32319118

[48] Gaebler C Wang Z Lorenzi JCC Muecksch F Finkin S Tokuyama M. Evolution of antibody immunity to SARS-CoV-2. Nature (2021) 591:639–44. doi: 10.1038/s41586-021-03207-w PMC8221082 33461210

[49] Muecksch F Wang Z Cho A Gaebler C Ben Tanfous T DaSilva J. Increased memory b cell potency and breadth after a SARS-CoV-2 mRNA boost. Nature (2022) . 607:128–34. doi: 10.1038/s41586-022-04778-y PMC9259484 35447027

[50] Hachmann NP Miller J Collier A-Y Ventura JD Yu J Rowe M. Neutralization escape by SARS-CoV-2 omicron subvariants BA.2.12.1, BA.4, and BA.5. Pro Nat Acad Sci USA (2022) . 387:86–8. doi: 10.1056/NEJMc2206576 PMC9258748 35731894

[51] Garcia-Beltran WF Lam EC St. Denis K Nitido AD Garcia ZH Hauser BM. Multiple SARS-CoV-2 variants escape neutralization by vaccine-induced humoral immunity. Cell (2021) . 184:2372–83. doi: 10.1016/j.cell.2021.03.013 PMC7953441 33743213

[52] Takada A Kawaoka Y. Antibody-dependent enhancement of viral infection: Molecular mechanisms and *In vivo* implications. Rev Med Biol (2003) . 13:387–98. doi: 10.1002/rmv.405 14625886

[53] Liu Y Soh WT Kishikawa JI Hirose M Nakayama EE Li S. An infectivity-enhancing site on the SARS-CoV-2 spike protein targeted by antibodies. Cell (2021) . 184:3452–3466.e18. doi: 10.1016/j.cell.2021.05.032 34139176 PMC8142859

[54] Liu Y Nakazaki Y Arase H. Infectivity-enhancing antibodies against SARS-CoV-2. Translat Regul Sci (2022) . 4:1–4. doi: 10.33611/trs.2021-021

[55] Naaber P Tserel L Kangro K Sepp E Jürjenson V Adamson A. Dynamics of antibody response to BNT162b2 vaccine after six months: A longitudinal prospective study. Lancet Region Health Europe (2021) 10:100208. doi: 10.1016/j.lanepe.2021.100208 PMC8418937 34514454

[56] Vallejo A Martín-Hondarza A Gómez S Velasco H Vizcarra P Haemmerle J. Cellular responses to membrane and nucleocapsid viral proteins are also boosted after SARS-CoV-2 spike mRNA vaccination in individuals with either past infection or cross-reactivity. Front Microbiol (2021) . 12:812729. doi: 10.3389/fmicb.2021.812729 35222312 PMC8874124

[57] Tang J Zeng C Cox TM Li C Son YM Cheon IS. Respiratory mucosal immunity against SARS-CoV-2 following mRNA vaccination. Sci Immunol (2022) eadd4853. doi: 10.1126/sciimmunol.add4853 35857583 PMC9348751

